# Association between hippocampal microglia, AD and LATE‐NC, and cognitive decline in older adults

**DOI:** 10.1002/alz.13780

**Published:** 2024-03-17

**Authors:** Alifiya Kapasi, Lei Yu, Sue E Leurgans, Sonal Agrawal, Patricia A Boyle, David A Bennett, Julie A Schneider

**Affiliations:** ^1^ Rush Alzheimer's Disease Center Rush University Medical Center Chicago Illinois USA; ^2^ Department of Pathology Rush University Medical Center Chicago Illinois USA; ^3^ Department of Neurological Sciences Rush University Medical Center Chicago Illinois USA; ^4^ Department of Psychiatry and Behavioral Sciences Rush University Medical Center Chicago Illinois USA

**Keywords:** Alzheimer's disease, cognitive decline, hippocampal microglia, LATE‐NC

## Abstract

**INTRODUCTION:**

This study investigates the relationship between microglia inflammation in the hippocampus, brain pathologies, and cognitive decline.

**METHODS:**

Participants underwent annual clinical evaluations and agreed to brain donation. Neuropathologic evaluations quantified microglial burden in the hippocampus, amyloid beta (Aβ), tau tangles, and limbic age‐related transactive response DNA‐binding protein 43 (TDP‐43) encephalopathy neuropathologic changes (LATE‐NC), and other common brain pathologies. Mixed‐effect and linear regression models examined the association of microglia with a decline in global and domain‐specific cognitive measures, and separately with brain pathologies. Path analyses estimated direct and indirect effects of microglia on global cognition.

**RESULT:**

Hippocampal microglia were associated with a faster decline in global cognition, specifically in episodic memory, semantic memory, and perceptual speed. Tau tangles and LATE‐NC were independently associated with microglia. Other pathologies, including Aβ, were not related. Regional hippocampal burden of tau tangles and TDP‐43 accounted for half of the association of microglia with cognitive decline.

**DISCUSSION:**

Microglia inflammation in the hippocampus contributes to cognitive decline. Tau tangles and LATE‐NC partially mediate this association.

## 1 BACKGROUND

Microglia are the brain's resident immune cells, accounting for 80% of immune cells and 10% to 15% of all cells found in the brain.^1^ In 1919, Pio Del Rio‐Hortega identified microglia and observed that these cells undergo morphologic transformation following brain tissue injury.^2^ It is now widely recognized that microglia are highly dynamic cells that are constantly surveilling the brain microenvironment, with the potential to display a profusion of morphologies. In Alzheimer ‘s disease (AD), microglia cells have been implicated in multifunctional roles in brain health and progression of disease, with studies suggesting a neuroprotective effect earlier in the disease process and a more neurotoxic effect in later stages.^3^ Further, the majority of risk genes for AD are highly expressed by microglia in the brain, including *APOE*,^4^ *BIN1*,^5^ *CD33*, and *TREM2*.^6^

Pathologic aging is a complex and heterogeneous process affected by many factors in the cellular environment. The hippocampal formation is vulnerable to the accumulation of AD and limbic age‐related transactive response DNA‐binding protein 43 (TDP‐43) encephalopathy neuropathologic changes (LATE‐NC),^7^, ^8^ and both disease processes are associated with microglia inflammation in the brain. Further, we and others have shown that both AD and LATE‐NC are potent determinants of late‐life cognitive decline.^9^ However, very few studies have examined the association of hippocampal microglia with cognitive decline in human‐based studies, and even fewer have examined the association of microglia with LATE‐NC. Because many of these pathologic processes are intertwined, the relationship between microglia, neurodegenerative pathologies, and cognition remains unclear and warrants further investigation.

In this study, we examined the association of microglia inflammation in the hippocampus, an early brain region vulnerable to both AD and LATE‐NC, with cognition, and the extent to which AD and LATE‐NC account for this association.

## 2 METHODS

### 2.1 Participants

Participants enrolled in two ongoing cohort studies of aging: the Religious Orders Study (ROS) and the Rush Memory and Aging Project (MAP).^10^ ROS started in 1994 and recruits older Catholic priests, nuns, and brothers from across the United States. MAP started in 1997 and recruits older adults from across the Chicagoland area. Eligibility for each study requires enrollment without known dementia, agreement to undergo annual clinical evaluation and interview, and consent to brain donation at the time of death. Follow‐up across all studies exceeds 85%, with autopsy rates exceeding 95% for ROS and 80% for MAP. Each study was approved by the Rush University Medical Center institutional review board. All participants signed an informed consent and Anatomical Gift Act for brain donation. The two studies share uniform structured neuropathologic evaluations. Age was calculated from birth date and death date. Education was based on self‐reported years of regular schooling. Sex was self‐reported.

**RESEARCH IN CONTEXT**

1. **Systematic review**: The authors reviewed the available literature about the association of microglia inflammation with cognitive decline and common age‐related pathologies. There are limited human‐based studies examining microglia inflammation in the hippocampus with longitudinal cognitive decline, and very little is known regarding the relationship between microglia, neurodegenerative pathologies, and cognition.
2. **Interpretation**: This study demonstrates that microglia inflammation in the hippocampus is an important driver of late‐life cognitive decline, above and beyond the presence of common neurodegenerative and cerebrovascular pathologies. Specifically, microglia inflammation was associated with decline in global cognition and in domains of episodic memory, semantic memory, and perceptual speed. Further, findings highlight that tau tangles and LATE‐NC partially mediated the association of microglia with cognitive decline.
3. **Future directions**: Further understanding of cellular interactions between microglia, neurons, and other glial and non‐glial cell types within brain microenvironments will provide important insight into regional heterogeneity in the brain.

### 2.2 Assessment of cognition

A standard uniform cognitive assessment was administered to each participant at baseline and at each annual follow‐up evaluation. These assessments employ a common battery of 19 neuropsychological tests and cover a broad range of cognitive abilities, including memory, attention, language, perception, and orientation. Seven tests of episodic memory, three of semantic memory, three of working memory, four of perceptual speed, and two of visuospatial ability were administered. A measure of global cognition was calculated by converting scores on individual tests to *z*‐scores using the baseline mean and standard deviation (SD) from all ROS and MAP participants and then averaging the *z*‐scores. Summary scores for each cognitive domain were derived by averaging the *z*‐scores of the neuropsychological tests specific to that cognitive domain, as previously described.^11^ The average clinical follow‐up was 11.7 years (SD = 6.87).

### 2.3 Neuropathology

Brain autopsies were performed following a standard protocol. Average *post mortem* interval was 11.8 h (SD = 11.5). One hemisphere was cut into 1‐cm coronal slabs and frozen. The contralateral hemisphere was cut into 1‐cm coronal slabs and fixed in 4% paraformaldehyde for at least 1 month. Paraformaldehyde fixed tissue from multiple brain regions was collected for various pathology data collection purposes, as described below.

#### 2.3.1 Hippocampal microglia

Microglia data collection in the hippocampus was added in 2018 on a consecutive subset of deceased and autopsied ROSMAP participants. Microglia data were collected across two hippocampal blocks: (1) the anterior hippocampus taken at the level of the uncus (*n* = 284 slides) and (2) the mid‐hippocampus taken at the level of the lateral geniculate nucleus (*n* = 284 slides). Immunohistochemistry for microglia was performed using HLA class II histocompatibility antigen antibodies (clone CR3/43; DakoCytomation, Carpinteria, CA; 1:100) on 6‐µm paraffin‐embedded sections. Three stages of microglia were evaluated based on morphology: stage 1 (multiple long ramified processes with a cell body diameter of <14 µm), with stage 1a included as a subtype of stage 1 (short, ramified processes with a cell body diameter of <14 µm); stage 2 (a cell body diameter >14 µm with thickened hypertrophic processes); and stage 3 (a cell body diameter >14 µm with a phagocytic, amoeboid morphology) (supplementary eFigure 1).

All stages of microglia morphology were evaluated in five separate hippocampal subregions: the proximal portion of CA1, the distal portion of CA1, the subiculum, the dentate gyrus from the mid‐hippocampus (supplementary Figure 1), and the CA1 subregion from the anterior hippocampus. Regions of interest were outlined using the StereoInvesitgator 8.0 software (version 2017). Counting frame parameters were set up to sample 15.0% of each subregion at 400× magnification. Different morphologic stages of microglia were marked in each counting frame. Counts were then upweighted by the stereology software to obtain the total number of microglia counts for each morphologic stage.

#### 2.3.2 AD pathology

A global AD pathology score was calculated from counts obtained using a modified Bielschowski stain from five brain regions, as previously described.^12^ Manual counts of neuritic and diffuse plaques and of neurofibrillary tangles in an area of greatest density from each region were used to determine Braak staging and Consortium to Establish a Registry for Alzheimer's Disease CERAD score. Beta‐amyloid positivity was evaluated in seven brain regions and used to determine Thal phase. A pathological diagnosis of AD was determined by the National Institute on Aging (NIA)‐Alzheimer's Association (AA) criteria.^13^ Separately, a total of eight brain regions, including the hippocampus, were assessed for cortical β‐amyloid and PHF‐tau tangle densities, as previously described.^14^, ^15^ For this study, analyses used the 8‐region composite measure for global β‐amyloid load and global tau‐tangle density. Measures of regional hippocampal tau tangles were used in pathway analyses.

#### 2.3.3 LATE‐NC

Phosphorylated transactive response DNA‐binding protein 43 (TDP‐43) pathology was identified by immunohistochemistry using a monoclonal antibody to phosphorylated TDP‐43 (pS409/410; 1:100). TDP‐43 was evaluated in eight brain regions;^16^ amygdala, hippocampus CA1 and subiculum, dentate gyrus, entorhinal cortex, anterior temporal pole, middle temporal gyrus, mid‐frontal gyrus, and orbitofrontal cortex, as described previously.^16^ Four distinct stages based on pathological distribution of TDP‐43 lesions were created; stage 0 (no TDP‐43 seen); stage 1 (TDP‐43 localized to the amygdala), stage 2 (deposition ofTDP‐43 in hippocampus and/or entorhinal cortex, but not in the neocortex), and stage 3 (deposition of TDP‐43 in the neocortex). Hippocampal sclerosis (HS) was evaluated in the mid‐hippocampus at the level of the lateral geniculate nucleus.^17^ HS was present if neuronal loss and gliosis in the CA1/subiculum subregion of the hippocampus was severe. For this study, the definition of LATE‐NC was dichotomized to include those with limbic or neocortical TDP‐43 pathology (ie, stage 2 or 3) with or without the presence of HS. Severity of TDP‐43 cytoplasmic inclusions in the hippocampus brain region was used as a regional measure in path analyses.

#### 2.3.4 Other age‐related pathologies

Other age‐related pathologies evaluated include macroscopic infarcts,^18^ microscopic infarcts,^19^ atherosclerosis, arteriolosclerosis,^20^ cerebral amyloid angiopathy,^21^ and Lewy bodies^22^ (supplement eMethods 1).

### 2.4 Statistical analyses

#### 2.4.1 Hippocampal microglia data

First, we examined the distributions of the total microglial density for each region using Q‐Q plots and summary statistics (ranges, quartiles, means, and skewness and kurtosis coefficients).

We then examined correlations across the five hippocampal subregions for total microglial density. All Spearman correlations were positive and strongly statistically significant (*p* < .001); the strongest correlation was between the proximal and distal CA1 subregions (rho = 0.87); the weakest correlation was between the anterior hippocampal CA1 and the dentate gyrus (rho = 0.40). We further examined the correlation structure using a principal component analysis (PCA). Only one component had an eigenvalue greater than 1; that component explained 69% of the total variance (supplement eTable 1). Therefore, we created a single composite measure for total hippocampal microglial density by scaling each regional density by dividing by the corresponding SD and then averaging the scaled densities. The composite measure for total microglia across all five subregions is well suited for standard statistical analyses (Supplement eFigure 2), that is, no transformation is required. Since every participant had some microglia in each subregion of the hippocampus, the resulting composite measure is always positive. Larger values correspond to a greater density of microglia.

We followed the same approach to summarize the densities of stage 2/3 microglia. Counts for stages 2 and 3 within each hippocampal subregion were divided by the area outlined in square millimeters to generate region‐specific densities. Unlike for the total microglia, data transformation was required for microglia stages 2/3. The logarithm corresponds to considering fold changes in stage 2/3 microglia. The PCA showed a single factor dominates (Supplement eTable 2). The log‐transformed regional measures were then scaled and averaged to obtain a composite measure across all five subregions for stage 2/3 microglia (Supplement eFigure 2). Participants with no stage 2/3 microglia will have a composite measure of 0, and greater stage 2/3 microglia densities result in higher composite measures. The composite measures for both total and stage 2/3 microglia were highly correlated (rho = 0.71, *p* < .001).

#### 2.4.2 Analytic approach

For this study, data review and PCA were used to derive composite summary measures for total microglia and stage2/3 microglia across five hippocampal subregions, as described above. Spearman correlation was used to examine bivariate analyses of hippocampal microglia with age and education. Analysis of variance (ANOVA) with Tukey's studentized range tests and *t* tests were used to compare the composite microglia measures by sex and by pathologic diagnoses. We used linear mixed‐effect models with random intercept and slope to examine the association of hippocampal microglia with rate of change in global cognition and, separately, change in each of the five cognitive domains. The longitudinal outcomes of interest were annual scores for global cognition and cognitive domains. The predictor of interest was the total hippocampal microglia burden. All models included terms for demographics and all pathology terms, time in years before death, and interactions between time and the other terms. The interaction term between time in years to death with microglial burden estimates the association of microglia with cognitive slopes. A significant and negative coefficient for the interaction suggests that a higher density of microglia is associated with a faster decline. Subsequent analyses employed similar mixed‐effect models replacing total microglia with stage 2/3 microglia. To examine neuropathologic associations with microglia, we employed linear regression models including with three terms for demographics (age, sex, and education) and nine pathology terms (cortical amyloid beta [Aβ], tau tangles, LATE‐NC, Lewy bodies, and cerebrovascular pathologies) with total microglia as the outcome, and separately with stage 2/3 microglia as the outcome. Subsequent models included interaction terms between AD and LATE‐NC. We employed path analysis to examine the extent to which the impact of hippocampal microglia on global cognitive decline could be attributable to AD and separately to LATE‐NC. In this model, we included regional hippocampal burden for tau‐tangle density and TDP‐43 cytoplasmic inclusion. The model partitions the total association effect of hippocampal microglia on cognitive decline into direct effect and indirect effects. Standardized path coefficients were used to estimate the proportions of indirect effects due to tau tangles and TDP‐43. Sensitivity analyses included linear regression models with global cognition as the outcome and hippocampal pathologies (microglia, tau, TDP‐43) as predictors (Supplementary eTable 3)

## 3 RESULTS

### 3.1 Participants

The demographic, clinical, and neuropathologic characteristics of our 284 participants are summarized in Table 1. On average, mean age at death was 91 years. The mean density of total hippocampal microglia was 2.11 (SD = 0.85), with values ranging from 0.02 to 5.42; the 25^th^ percentile was 1.58, the 50th percentile was 2.00, and the 75th percentile was 2.62. Microglia in stage 2/3 were present in 90% of hippocampi, with 85% having stage 2 and 70% stage 3. The mean density of stage2/3 microglia (after log transformation) was 0.44 (SD = 0.37), with values ranging from 0 to 1.65.

**TABLE 1:** Characteristics of participants.

	Mean (SD) or *n* (%)	
***Demographics***		
Age at death, years	91.4 (6.40)	
Sex, women	201 (71)	
Education, years	16.1 (3.59)	
Clinical follow‐up	11.7 (6.87)	
***Cognitive score***	*Baseline*	*Last visit*
Global cognition	0.08 (0.60)	−0.96 (1.19)
Episodic memory	0.09 (0.76)	−0.83 (1.38)
Semantic memory	0.09 (0.83)	1.13 (1.42)
Working memory	0.06 (0.83)	−0.71 (1.12)
Perceptual speed	0.08 (0.80)	−1.06 (1.04)
Visuospatial ability	0.14 (0.81)	−0.45 (1.06)
***Neuropathologic***		
Density of total microglia	2.11 (0.85)	
Density of stage 2/3 microglia	0.44 (0.37)	
AD pathologic diagnoses (NIA‐AA)	181 (64%)	
Global AD pathology score	0.66 (0.58)	
Global Aβ load	0.36 (0.42)	
Global tau‐tangle density	1.79 (1.52)	
Hippocampal tangle density	4.56 (2.34)	
LATE‐NC		
Stage 0	130 (46%)	
Stage 1	45 (16%	
Stage 2	31 (11%)	
Stage 3	78 (27%)	
Hippocampal TDP‐43 pathology score	3.19 (6.26)	
Lewy bodies	85 (30%)	
Macroscopic infarcts (chronic)	101 (36%)	
Microscopic infarcts (chronic)	124 (44%)	
Arteriolosclerosis, moderate to severe	58 (20%)	
Atherosclerosis, moderate to severe	72 (25%)	
Cerebral amyloid angiopathy, moderate to severe	106 (37%)	

Neurodegenerative pathologies were common, with a pathological diagnosis of AD present in 64%, LATE‐NC (stages 2 and 3) in 38%, and Lewy bodies in any brain region in 30%. In bivariate analyses, age at death was positively associated with hippocampal microglia, rho = 0.29, *p* < .001, but not with education (*p *= .41), and it did not differ by sex (*p *= .65). Total hippocampal microglia were correlated with tau tangles (rho = 0.43, *p* < .001), cortical Aβ burden (rho = 0.13, *p* = .03), LATE‐NC (*t* value = −8.50*, p* < .001), and atherosclerosis (*F* test = 4.92, *p* < .002). Hippocampal microglia were not associated with macro‐ or microscopic infarcts, arteriolosclerosis, or the presence of Lewy bodies (all *p* > .07).

### 3.2 Hippocampal microglia with cognition

**FIGURE 1:**
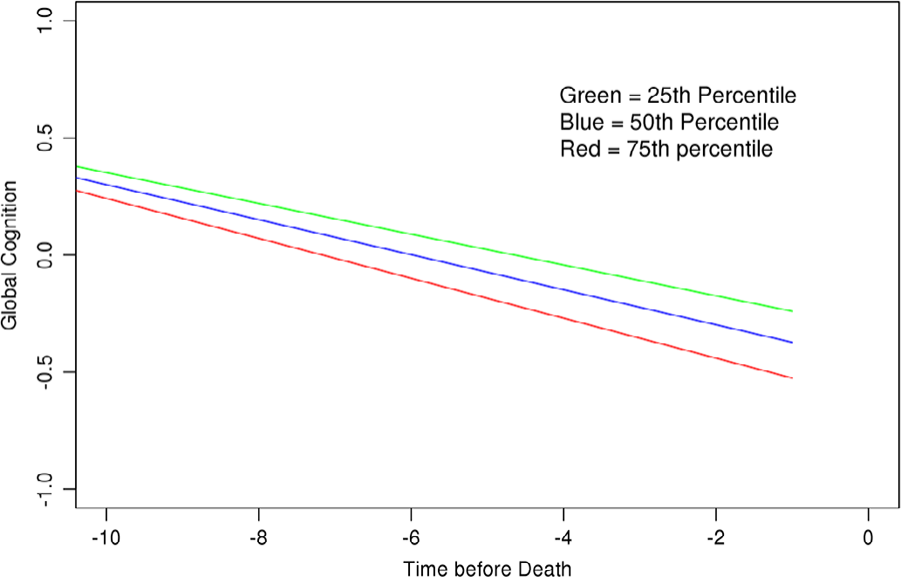
Association of hippocampal microglia pathology with decline in global cognition. Derived from a mixed‐effects model in Table 3. Percentile refers to 25th, 50th, or 75th percentile of total microglia densities.

In longitudinal analyses, total microglia burden was associated with a faster decline of 0.020 unit per year in global cognition (Figure 1), 0.026 unit per year in episodic memory, 0.020 unit per year in semantic memory, and 0.012 unit in perceptual speed (Table 2). As expected, tau tangles and LATE‐NC were both independently associated with global cognitive decline (estimate = −0.03; SE = 0.004; *p* < .001 and estimate = −0.02; SE = 0.01;*p* = .03, respectively). In contrast, Aβ pathology was not associated with cognitive decline (estimate = 0.09; SE = 0.08; *p* = .27), with tau tangles included in the same model.

**TABLE 2:** Association of microglia with level and decline in global cognition and cognitive domains.

	Total microglia estimate (SE, *p* value)	Stage 2/3 microglia estimate (SE, *p*‐value)
**Global cognition**		
*Level*	−0.306 (0.07, < .001)	−0.263 (0.17, .13)
*Decline*	−0.020 (0.01, < .001)	−0.008 (0.01, .59)
**Episodic memory**		
*Level*	−0.449 (0.09, < .001)	−0.384 (0.22, .08)
*Decline*	−0.026 (0.01, < .001)	−0.017 (0.02, .32)
**Semantic memory**		
*Level*	−0.327 (0.09, < .001)	−0.290 (0.21, .17)
*Decline*	−0.020 (0.01, .003)	−0.016 (0.02, .37)
**Working memory**		
*Level*	−0.086 (0.07, .19)	−0.001 (0.16, 1.00)
*Decline*	−0.008 (0.01, .08)	0.007 (0.01, .58)
**Perceptual speed**		
*Level*	−0.193 (0.08, .01)	−0.275 (0.19, .15)
*Decline*	−0.012 (0.006, .04)	−0.002 (0.02, .87)
**Visuospatial **		
*Level*	−0.045 (0.07, .54)	−0.035 (0.18, .85)
*Decline*	−0.006 (0.01, .27)	0.002 (0.01, .87)

*Note*: Coefficients of microglia scores from mixed‐effects regression models of longitudinal cognitive outcomes adjusted for age, male sex, education, global AD pathology score, LATE‐NC, macro‐ and microscopic infarcts, atherosclerosis, arteriolosclerosis, cerebral amyloid angiopathy, and Lewy bodies, interactions of all terms with time, and time expressed as years before death.

Because prior studies showed that morphologic subtypes of microglia might be important in pathologic aging, secondary analyses examined the association of stage 2/3 microglia with cognition (proximate‐to‐death and decline). We found that stage 2/3 microglia were not associated with rate of decline in global cognition or any cognitive domain. Further, no associations with cognition proximate‐to‐death were seen (Table 2).

### 3.3 Hippocampal microglia, AD, and LATE‐NC

**FIGURE 2:**
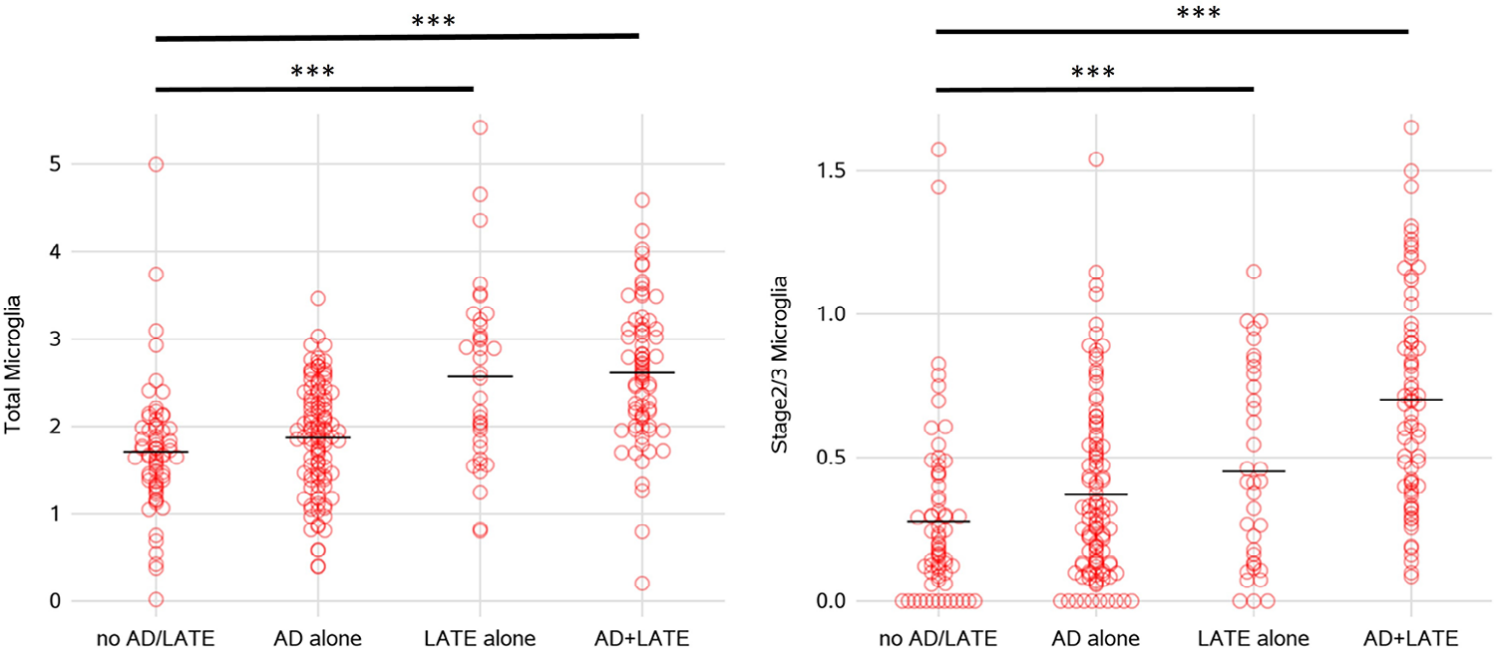
Burden of hippocampal microglia by combination of AD and LATE‐NC pathologic diagnoses. Pathologic AD was defined as either intermediate or high likelihood of AD using NIA‐AA criteria; LATE‐NC was defined as having limbic or neocortical TDP‐43 pathology (stage 2 or 3). Statistical significance of *p* < .001 across groups are denoted by three asterisks.

Because both AD and LATE‐NC commonly involve the hippocampus, we examined the burden of microglia across four pathologic groups: (1) no AD or LATE‐NC (*n* = 67, 24%), (2) AD without LATE‐NC (*n* = 108, 38%), (3) LATE‐NC without AD (*n* = 36, 13%), and (4) mixed AD/LATE‐NC (*n* = 73, 26%). Total microglial density differed across the four groups (*p* < .001). Compared to group 1 (without AD or LATE‐NC,) the burden of total microglia was higher among LATE without AD (*t* = −4.44, *p* < .001) and mixed AD/LATE‐NC groups (*t* = −6.95, *p* < .001), but not AD without LATE (t = −1.49, *p* = .14), suggesting heterogeneity in hippocampal microglia burden across isolated and mixed pathologic groups. The burden for stage 2/3 microglia across pathologic groups showed similar patterns (Figure 2).

Next, we examined the association of common age‐related pathologies with total microglia burden. In linear regression models with terms for age at death, sex, education, and common age‐related neuropathologies, tau tangles and LATE‐NC were independently associated with a higher burden of total microglia. Other age‐related pathologies, including Aβ and cerebrovascular pathologies, were not associated with total microglia burden (Table 3). Secondary analyses examined the association of stage 2/3 microglia with common age‐related pathologies and obtained similar findings (Table 3).

**TABLE 3:** Association of common age‐related neuropathologies with hippocampal microglia.

Pathologic Indices	Total microglia estimate (SE, *p* value)	Stage 2/3 microglia estimate (SE, *p* value)
Global Aβ load	−0.160 (0.11, .16)	0.007 (0.05, .89)
Global tau‐tangle density	0.166 (0.04, <.001)	0.093 (0.01, <.001)
LATE‐NC	0.232 (0.04, <.001)	0.080 (0.02, <.001)
Lewy bodies	−0.0003 (0.10, 1.00)	0.015 (0.04, .72)
Gross infarcts	0.022 (0.10, .82)	0.043 (0.04, .31)
Microscopic infarcts	−0.038 (0.09, .68)	0.036 (0.04, .37)
Arteriolosclerosis	−0.013 (0.05, .81)	−0.031 (0.02, .18)
Cerebral amyloid angiopathy	−0.043 (0.05, .35)	0.008 (0.02, .67)
Atherosclerosis	0.122 (0.08, .09)	0.047 (0.03, .13)

*Note*: Estimates derived from linear regression models adjusted for age at death, male sex, education.

Subsequent analyses investigated the association of tau tangles with total microglia burden across LATE‐NC stages: no interaction was seen (*p* = .90). Pathologic findings of HS were noted in 9% of participants. Because LATE‐NC, in this study, is defined as having TDP‐43 pathology (stage 2/3) with or without HS, we further adjusted for HS. In these models, findings remained unchanged, with TDP‐43 pathology independently associated with total microglia burden (estimate = 0.10; SE = 0.04, *p *= .01). In addition, HS was strongly associated microglia burden (estimate = 0.60; SE = 0.08, *p* < .001).

### 3.4 Path analyses

**FIGURE 3:**
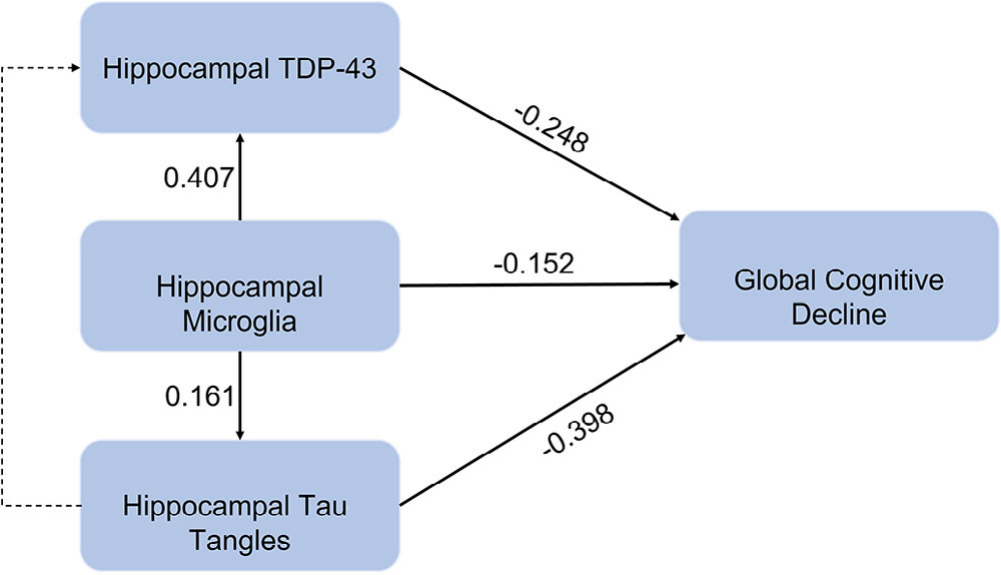
Association of hippocampal microglia on global cognition considering mediation by regional hippocampal tau tangles and TDP‐43. Path coefficients derived from path analysis. Each single‐headed arrow denotes a hypothesized unidirectional effect of one variable on another. Paths that were statistically significant at *p* < .05 are represented by solid lines. A path that was hypothesized but not statistically significant is denoted by dashed lines.

Employing path analyses, we tested for the direct effect of microglia on cognitive decline cognition, as well as the indirect effect of microglia on cognition through tau tangles and TDP‐43 pathology. Because we were interested in the regional effects of microglia in the hippocampus on global cognitive decline, we used regional data for tau tangles and TDP‐43 pathology from the hippocampus. The analyses revealed two significant pathways: (1) microglia with cogntive decline thought regional tau tangles and (2) microglia with cognitive decline through regional TDP‐43. A third pathway of microglia with cogntive decline through regional tangles and regional TDP‐43 was not established (Figure 3).

**FIGURE 4:**
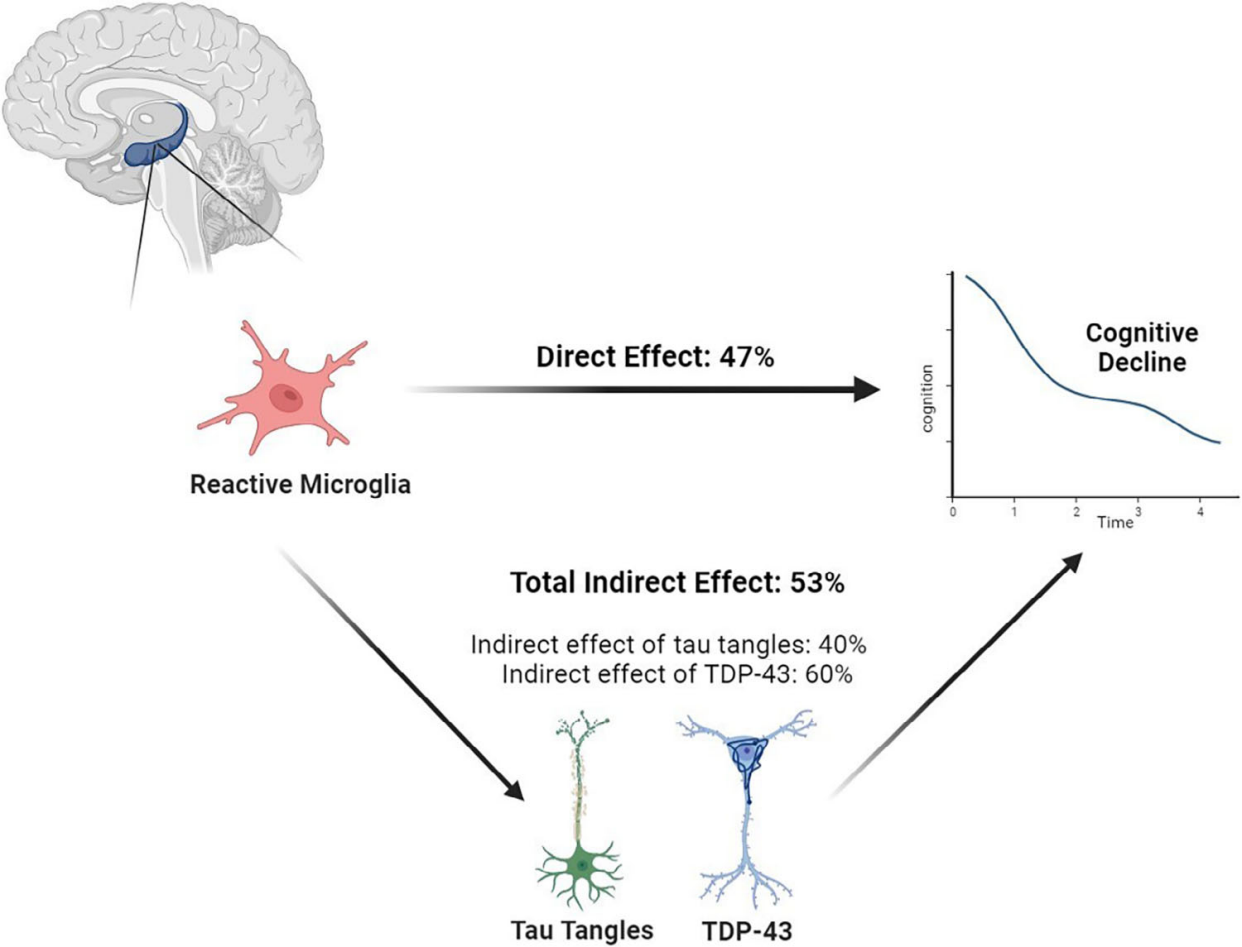
Schematic representation linking microglia inflammation, AD, and LATE‐NC with cognitive decline. Figure created with Biorender.com.

The total standardized effect of total microglia on global cognitive decline was −0.32 (SE = 0.06, *p* < .001). About half of the total effect represented a direct effect of total microglia on decline (estimate = −0.15; SE = 0.06, *p* = .011). The remaining half was due to indirect effects via both tangles and TDP‐43 pathology (estimate = −0.17, SE = 0.04, *p* < .001). Of the indirect effect, TDP‐43 accounted for 60%, and tangles accounted for about 40% of it (Figure 4).

## 4 DISCUSSION

In this study of 284 community‐based participants, we examined the association of microglia inflammation in the hippocampus with cognitive decline and the relationship with AD and LATE‐NC pathology. We found that microglia inflammation in the hippocampus was an important driver of late‐life cognitive decline, above and beyond the presence of common neurodegenerative and cerebrovascular pathologies. Specifically, we found that total microglia burden was associated with decline in global cognition and in domains of episodic memory, semantic memory, and perceptual speed. Compared to participants with no AD/LATE, hippocampal microglia burden was higher among those with LATE‐NC (‐AD) and mixed AD/LATE‐NC, but not AD (‐LATE). Microglial burden was independently associated with LATE‐NC and with tau tangles, but not Aβ pathology. Regional hippocampal measures of TDP‐43 and tau tangles combined accounted for half of the total association between microglia and global cognition, and, interestingly, TDP‐43 pathology contributed more than AD (tau) pathology to the association between microglia and global cognition. Together, these findings provide compelling evidence that complex pathologic processes within the hippocampus contribute to cognitive dysfunction in old age.

This study extends prior work in several ways. First, very little is known about the contribution of microglia inflammation in the brain with late‐life cognitive decline. Prior studies examining longitudinal cognitive profiles showed that AD, LATE‐NC, Lewy bodies, and cerebrovascular diseases were all important contributors to cognitive decline, with AD and LATE‐NC having the most potent effect.^23^ In this study, we report a larger proportion of the effect of microglia on cognitive decline being direct. This important finding highlights that microglia inflammation, particularly in the hippocampus, is a separate and distinct biological process that contributes to cognitive decline in older adults that is not fully explained by the presence of other neurodegenerative and cerebrovascular pathologies. Supporting this, other studies demonstrated that neuroinflammation in the anterior temporal brain structurers measured by positron emission tomography^24^ and soluble TREM2 evaluated by cerebrospinal fluid^25^ was associated with longitudinal cognitive change. Surprisingly, we found no association between stage 2/3 microglia with level or decline in global cognition or any cognitive domain, a finding that may be the result of having relatively lower amounts of stage 2/3 microglial cells. In addition to neuropathologic contributors, many environmental and/or biological factors could be driving or modulating microglia inflammation. There is substantial evidence now implicating lifestyle/psychosocial stressors^26^, ^27^, ^28^ (eg, economic status, adverse life events, access to educational and healthcare resources, geographical factors, and others) with brain health in older adults. Additional lifestyle factors, including poor or fragmented sleep and dietary habits,^29^, ^30^ have also been shown to influence microglia inflammation. More recent work implicates other biological mechanisms, including dysbiosis of the gut^31^, ^32^ and the role of the inflammasome, innate immune sensors that regulate inflammation in response to harmful stimuli.^33^ Together, these studies solidify the fact that cognitive aging is a complex disease process and that much work remains to understand the relationship between lifestyle stressors/factors, microglia inflammation, and cognition.

Second, microglia pathology in the context of LATE‐NC is largely unexplored. This study demonstrates that microglia inflammation in the hippocampus is associated with LATE‐NC and that regional TDP‐43 pathology accounts for a larger proportion of the effect between microglia and cognition than regional tangles. Notably, we found that the effects of microglia on cognition worked through two separate pathways, one through TDP‐43 and the other through tangles; however, we acknowledge that these relationships are complex and most likely bidirectional. LATE‐NC is determined by the accumulation of TDP‐43 cytoplasmic inclusions in predominantly mesial temporal brain regions. On *post mortem* brain tissue, TDP‐43 inclusions can be observed in both neurons and glial cells.^34^ Prior studies using brain tissue from cases with frontotemporal lobar degeneration, amyotrophic lateral sclerosis, and AD show that TDP‐43 inclusions can be localized in both astrocytes and oligodendrocytes.^35^ Recent experimental findings from animal model studies demonstrated that astrocytic TDP‐43 dysfunction in the hippocampus contributed to progressive memory loss through aberrant chemokine signaling.^36^ Genetic factors may also link microglia and TDP‐43. A recent study showed mechanistic links between microglia and TDP‐43 demonstrating that microglia use TREM2 signals to respond to TDP‐43 pathology.^37^ Given the substantial role of glia pathology in neurodegenerative diseases, further work to expand on cell‐type vulnerability to TDP‐43 pathology and associated genetic or signaling pathways will be important to understand.

Lastly, there is substantial evidence suggesting that microglia proliferation and activation may have a central pathogenic role in AD, with studies suggesting a dynamic interplay between microglia and Aβ plaques.^38^, ^39^ In the current study, we did not find an association between microglia burden and global Aβ pathology burden. One plausible explanation may be that we are quantifying microglia pathology in the hippocampus, a brain region that does not accumulate a high burden of Aβ neuritic/cored plaques. This observation leads to the question of whether there is phenotypic heterogeneity in the presentation of microglia cells across different brain regions (such as hippocampus vs neocortical).

Microglia are enigmatic cells. They are dynamic and have diverse functions that can both benefit and damage the brain. In pathologic aging, microglial cells can be influenced by multiple stressors, including deregulated signaling, pathologic burden, and cellular changes within the microenvironment. A recent experimental study described how microglia cells in the hippocampus decreased expression of “environment sensing” genes, thereby predisposing the hippocampus to being more vulnerable to aging and deposition of proteinopathy.^40^ One hypothesis is that the accumulation of pathologic burden in the brain stresses microglia cells, which may be a driving factor in how these cells transition between homeostatic and senescent phenotype and that the accumulation of senescent microglia sustains and drives build‐up of pathologic burden.^41^ Interestingly, microglia have also been implicated in the propagation and spread of tau pathology through anatomically connected neurons within the hippocampus.^42^ In line with this, other studies using human brain tissue have reported that changes in glial phenotypes in the cortex and white matter brain regions represent an early phenomenon that precedes overt tau deposition and likely contributes to cell damage.^36^ Understanding cellular interactions between neurons, microglia, and other glial and non‐glial cell types within “local” brain microenvironments remains elusive, and studies directed toward this will provide important insights into regional heterogeneity in the brain. In addition, given that microglia also exert neuroprotective effects, future studies directed at understanding how microglia combat cellular stress and contribute to cognitive resilience and brain reserve will be important.

With the advent of AD biomarkers and disease‐modifying therapies, understanding disease heterogeneity, mixed pathologies, and brain structural changes over time is of critical importance. Hippocampal volume is often used to represent neurodegeneration (N) in the Amyloid/Tau/Neurodegeneration (ATN) criteria.^43^ The hippocampus is vulnerable to the accumulation of varied pathologic processes, including AD, LATE‐NC,^8^ cerebrovascular pathologies,^44^, ^45^ microstructural tissue integrity,^46^ and primary age‐related tauopathy.^47^ Examining the contribution of microglia inflammation to hippocampal structure will be important, especially in the context of mixed pathologies, as well as whether lifestyle stressors and/or other factors contribute to these pathways.

This study has strengths and weaknesses. The limitations of this study include a lack of double staining experiments to examine direct co‐expression relationships, subjectivity in manual identification of microglia, our inability to distinguish between other central nervous system‐associated macrophages or possible infiltrating monocytes, and the fact that study participants were largely non‐Hispanic White and highly educated, characteristics that differ from the general population. Strengths are that data came from well‐characterized older persons recruited from the community and followed with longitudinal cognitive evaluations and detailed neuropathologic evaluation. Our findings underscore the complexity of pathologic processes occurring in the hippocampus that contribute to late‐life cognitive decline.

## AUTHOR CONTRIBUTIONS

Research project: Alifiya Kapasi, Lei Yu, Julie A Schneider were involved in the conception, design, and execution of the project. Manuscript: Alifiya Kapasi wrote the first draft, and Lei Yu, Sue E Leurgans, Sonal Agrawal, Patricia A Boyle, David A Bennett, and Julie A Schneider reviewed and critiqued subsequent drafts.

## CONFLICTS OF INTEREST STATEMENT

JAS serves as a consultant for Cervau Technologies (now Lantheus) and Meilleur Technologies. AK, LY, SEL, SA, PAB, and DAB have no relevant disclosures. Author disclosures are available in the supporting information.

## CONSENT STATEMENT

All human subjects provided informed consent

## Supporting information

Supporting Information

Supporting Information
