# Supplementary material for: Association between hippocampal microglia, AD and LATE‐NC, and cognitive decline in older adults
Source: Alzheimers Dement. 2024 Mar 17;20(5):3193–202. doi: 10.1002/alz.13780 (PMC11095444; doi:10.1002/alz.13780)
Supplement: Supplementary file 1 — Supporting Information [file ALZ-20-3193-s002.docx]

**Supplementary Material**

**eFigure 1. Hippocampal subregions and microglia morphology**


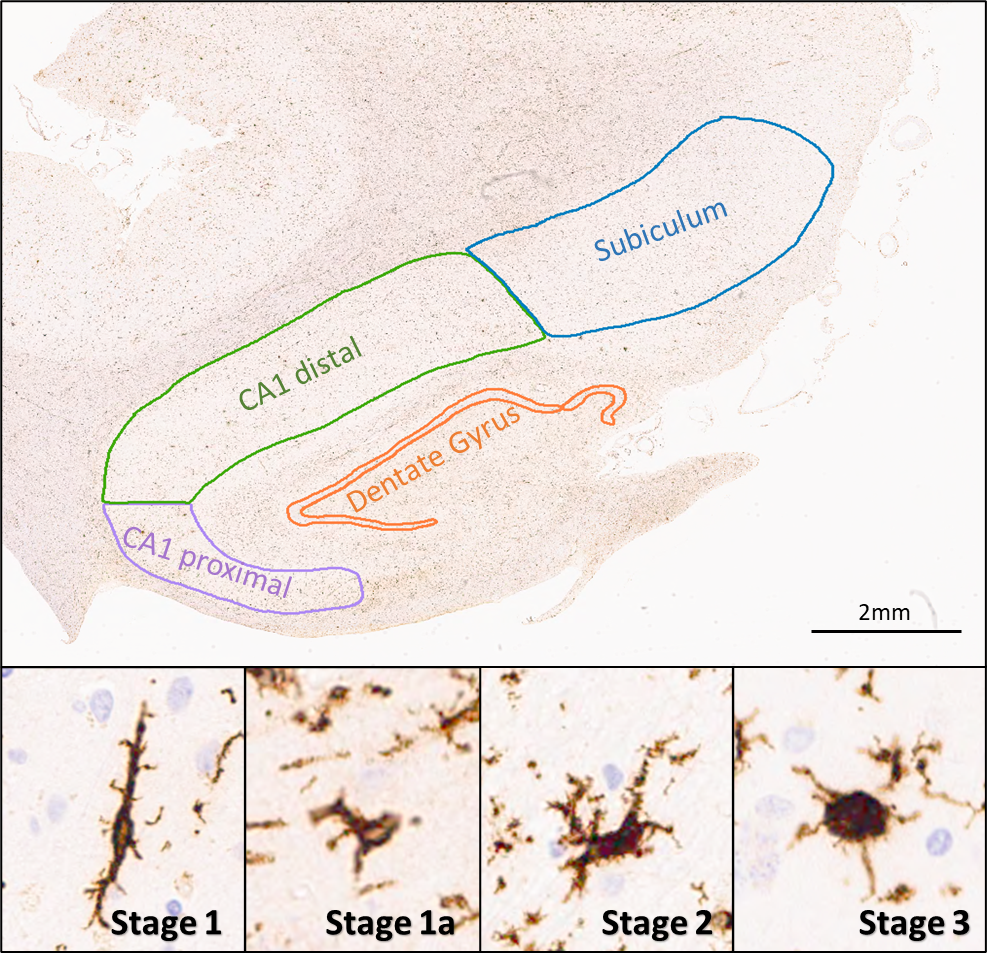


CR3-43 antibody stain of the mid hippocampus depicting each subregion of interest and morphologic stages of microglia.

**eFigure 2. Q-Q plots of summary measures for total microglia and stage 2/3 microglia across the hippocampus**


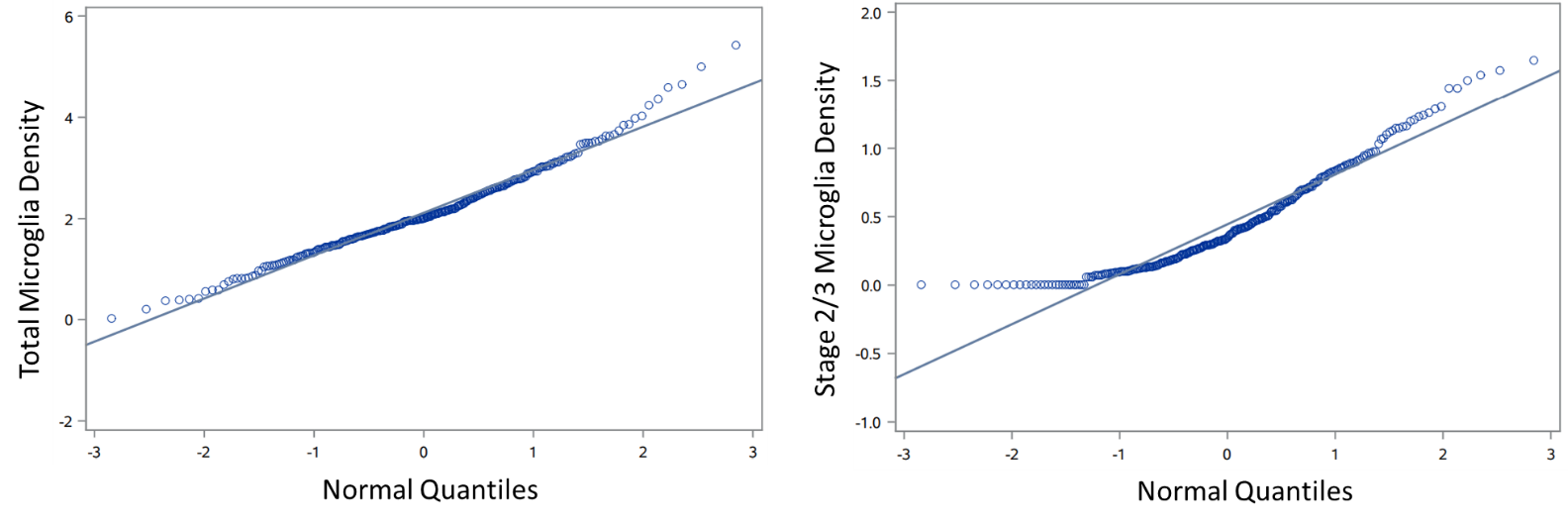


**eMethods 1. Description of other age-related pathologies**

*Atherosclerosis:* Large vessel atherosclerosis was evaluated at the circle of Willis at the base of the brain, and included examination of the vertebral, basilar, posterior, middle, and anterior cerebral arteries, and their proximal branches. Visual examination included the number of atherosclerotic plaques, extent of involvement in each vessel, and the degree of vessel occlusion. For analyses, a semi-quantitative 4-level grading system (0-3) was used.

*Arteriolosclerosis:* Small vessels in the basal ganglia were evaluated on hematoxylin and eosin-stained sections as none, mild, moderate, or severe.

*Cerebral Amyloid Angiopathy (CAA):* Meningeal and parenchymal vessels from 4 neocortical regions (midfrontal, midtemporal, inferior parietal, and calcarine cortices) were evaluated on sections immunostained with monoclonal antibodies against Aβ, 4G8 (1:9000; Covance Labs, Madison, WI). CAA was scored as none (0), mild (1), moderate (2), and severe (3).

*Cerebral Infarcts:* Location, age, and size of macroscopic infarcts visible on gross examination were documented. Subsequently, the age of macroscopic infarct was confirmed by microscopy. Microscopic infarcts were not visible to the naked eye and were identified by microscopy in tissue blocks, including cortical, subcortical, midbrain, and cerebellum regions. Location and age were documented. Chronic infarcts were considered for analyses, and all infarct variables were categorized into absent vs. present.

*Lewy body pathology*: Lewy bodies were evaluated using a phosphorylated α-synuclein antibody (Wako; 1:20,000) in seven regions including substantia nigra, limbic and neocortical regions.

**eTable 1. Factor loadings for total microglia across 5 subregions of the hippocampus**

|  | Factor Pattern: Factor1 |
| --- | --- |
| CA1 distal segment (mid-hip) | 0.92646 |
| CA1 proximal segment (mid-hip) | 0.89364 |
| Subiculum (mid-hip) | 0.87167 |
| CA1 subregion (anterior hip) | 0.81128 |
| Dentate gyrus (mid-hip) | 0.62740 |

**eTable 2. Factor loadings for microglia stage2/3 across 5 subregions of the hippocampus**

|  | Factor Pattern: Factor1 |
| --- | --- |
| CA1 distal segment (mid-hip) | 0.89085 |
| CA1 proximal segment (mid-hip) | 0.84237 |
| Subiculum (mid-hip) | 0.74212 |
| CA1 subregion (anterior hip) | 0.69562 |
| Dentate gyrus (mid-hip) | 0.66173 |

**eTable 3.**

|  | Global Cognition, proximate to death | | |
| --- | --- | --- | --- |
|  | Model 1 | Model 2 | Model 3 |
| Hippocampal total microglia burden | -0.54  (0.08, <0.001) |  | -0.36  (0.08, <0.001) |
| Hippocampal tangle burden |  | -0.18  (0.028, <0.001) | -0.17  (0.03, <0.001) |
| Hippocampal TDP-43 burden |  | -0.06  (0.01, <0.001) | -0.04  (0.01, <0.001) |

Linear regression models adjusted for age at death, sex, and education with global cognition as the outcome. Model 1 demonstrates β co-efficient for hippocampal microglia as the predictor. Model 2 shows β co-efficient for when hippocampal tangles and TDP-43 burden both predictors. Model 3 shows β co-efficient for when all 3 hippocampal pathologies are in the model. Values in cells are β-coefficient estimate (SE, *p*-value).
